# Supplementary material for: A Novel Recurrent 200 kb CRYL1 Deletion Underlies DFNB1A Hearing Loss in Patients from Northwestern Spain
Source: Genes (Basel). 2025 May 30;16(6):670. doi: 10.3390/genes16060670 (PMC12191918; doi:10.3390/genes16060670)
Supplement: Supplementary file 1 [file genes-16-00670-s001.zip › Supplementary Figure 1 CRYL1 20250507.pdf]

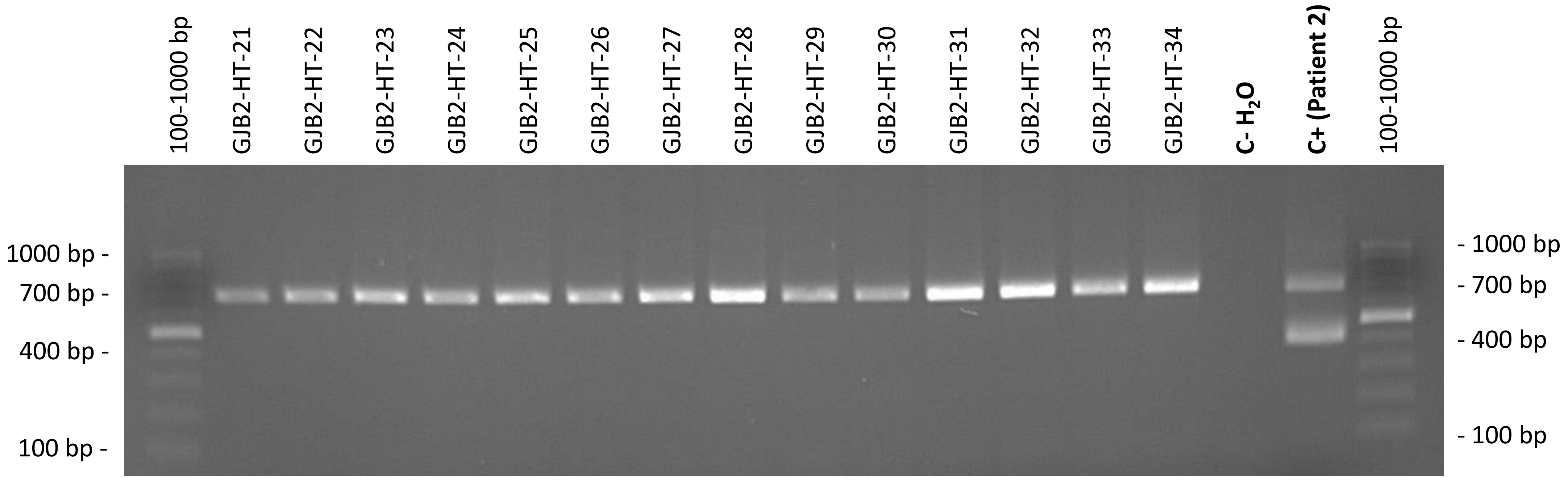

Supplementary Figure 1. Absence of del(200kb)insATTATA in patients analyzed with versions of the OTOgenics^{TM} platform designed to detect *CRYL*1 deletions and that had been found to be carriers of monoallelic pathogenic *GJB2* variants, based on results from the NGS panel

Agarose gel electrophoresis of products from triple-primer PCR designed to screen for del(200kb)insATTATA in 14 deaf patients previously evaluated in our laboratory with versions of the OTOgenics^{TM} platform that contained probes for detection of *CRYL1* deletions and who were single heterozygotes for a pathogenic/likely pathogenic *GJB*2 variant. As predicted from the OTOgenics^{TM} test results, none of them contained the 436 bp band specific for del(200kb)insATTATA. A 702 bp fragment is obtained from the allele without the *CRYL1* deletion. Patient #2 sample was included as a positive control.

Supplementary Figure 1
